# Investigation on the Peroxidase-like Activity of Vitamin B6 and Its Applications in Colorimetric Detection of Hydrogen Peroxide and Total Antioxidant Capacity Evaluation

**DOI:** 10.3390/molecules27134262

**Published:** 2022-07-01

**Authors:** Chun-Yan Zhang, Li-Jing Peng, Guo-Ying Chen, Hao Zhang, Feng-Qing Yang

**Affiliations:** School of Chemistry and Chemical Engineering, Chongqing University, Chongqing 401331, China; 20155520@cqu.edu.cn (C.-Y.Z.); 20165614@cqu.edu.cn (L.-J.P.); 201918021143@cqu.edu.cn (G.-Y.C.); 20161802127@cqu.edu.cn (H.Z.)

**Keywords:** vitamin B6, peroxidase-like activity, colorimetric detection, hydrogen peroxide, gallic acid

## Abstract

The peroxidase-like activity of vitamin B6 (VB6) was firstly demonstrated by catalyzing the peroxidase chromogenic substrate 3,3′,5,5′-tetramethylbenzidine (TMB) at the existence of H_2_O_2_. The influence of different factors on the catalytic property of VB6, including pH, temperature, VB6 concentration, and incubation time, were investigated. The steady-state kinetic study results indicate that VB6 possesses higher affinity to H_2_O_2_ than natural horseradish peroxidase and some other peroxidase mimics. Besides, the radical quenching experiment results confirm that hydroxyl radical (•OH) accounts for the catalytic process. Based on the excellent peroxidase-like catalytic activity of VB6, the colorimetric methods for H_2_O_2_ and gallic acid (GA) detection were developed by measuring the absorbance variance of the catalytic system. Under the optimal conditions, the linear ranges of the methods for H_2_O_2_ and GA determination with good selectivity are 50.0–600.0 μM and 10.0–50.0 μM, respectively. In addition, the developed method was applied in the detection of H_2_O_2_ in milk samples and evaluation of total antioxidant capacity of different tea infusions. This study may broaden the application prospect of VB6 in environmental and biomedical analysis fields, contribute to profound insight of the physiological functions of VB6, as well as lay foundation for further excavation of small-molecule peroxidase mimics.

## 1. Introduction

Horseradish peroxidase plays a significant role in organisms for its high catalytic performance and excellent substrate selectivity, which has been extensively applied in various biosensors for the determination of H_2_O_2_, glucose, glutathione, dopamine, and so on [1,2,3,4]. Nevertheless, the application of natural horseradish peroxidase is limited by some inherent drawbacks such as high cost of preparation process, nonreusability, weak stability towards variable temperature and pH, which may also lead to the undesirable inaccuracy of the determination results [5,6]. Although several immobilization methods were used to enhance their durability under harsh environmental conditions, the catalytic activities of enzymes are always limited by the biocompatibility of the support materials, inevitable enzyme activity loss during the immobilization and limited enzyme loading amount [7,8]. Therefore, it is an intriguing topic to develop peroxidase mimics as an alternative to the natural enzyme. Recently, considerable effort has been contributed to constructing nanomaterials-based peroxidase mimics, such as metallic oxides and salts [9,10,11], some noble metals, including Pt [12], Pd [13], and Au@Pt nanoparticles [14], layered double hydroxides [15], and quantum dots [16,17]. However, the large-scale application of nanomaterials-based enzyme mimics is hampered by several constraints, such as environmental pollution, weak enzyme-like activity, poor biocompatibility, heavy reliance on the skilled technician and sophisticated apparatus, and unavoidable enzyme-like activity variance between batches [18,19].

Recently, small molecules as peroxidase simulants have attracted tremendous attentions due to their easy commercial availability, good biocompatibility, and excellent homogeneous catalysis capacity. Since Liu et al. [20] discovered the peroxidase mimicking property of fluorescein, a series of small-molecule peroxidase mimics such as fluorescein derivatives [21,22], acidic amino acids [23], guanosine triphosphate (GTP) [24], ethylene diamine tetraacetic acid (EDTA) [25], adenine phosphate [26], and vitamin B3 [18] were successively reported possessing catalytic ability like natural peroxidase. Based on the outstanding catalytic activity, the small molecules with peroxidase-like property were extensively utilized to fabricate diverse sensors for the detection of bioactive substances, antioxidant capacity evaluation, and enzyme-linked immunoassay with high sensitivity and selectivity.

Vitamin B6 (VB6), as one of the water-soluble vitamins, is naturally present in various foods and plays a significant role in the synthesis of serotonin, and the deficiency in VB6 may lead to muscle weakness, irascibility, depression, and short-period amnesia [27]. Although the vital physiological actions of VB6 have been investigated extensively, there is still no report to demonstrate its excellent catalytic activity as peroxidase mimic. Herein, the peroxidase-like activity of VB6 was investigated in this study using the classical peroxidase substrate 3,3′,5,5′-tetramethylbenzidine (TMB) to obtain the blue product TMB oxides (oxTMB) in the presence of H_2_O_2_. Moreover, based on signal response variance of the VB6-TMB-H_2_O_2_ reaction system, a facile colorimetric method for H_2_O_2_ and gallic acid (GA) determination was established. With the increasing amount of GA addition, the blue oxTMB fades gradually due to its strong reducibility, and thereby resulting in the decreased readout signal. The developed method was further utilized in H_2_O_2_ detection in milk samples and total antioxidant capacity evaluation of tea infusion. This study may facilitate profound insight of the physiological action of VB6 and give inspirations for excavating more small-molecule enzyme mimics.

## 2. Results and Discussion

### 2.1. Peroxidase-like Catalytic Activity of VB6

**Figure 1:**
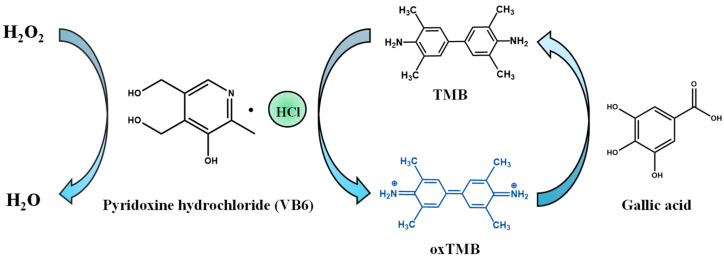
Schematic illustration of peroxidase-like activity of VB6 and the detection mechanism of gallic acid.

**Figure 2:**
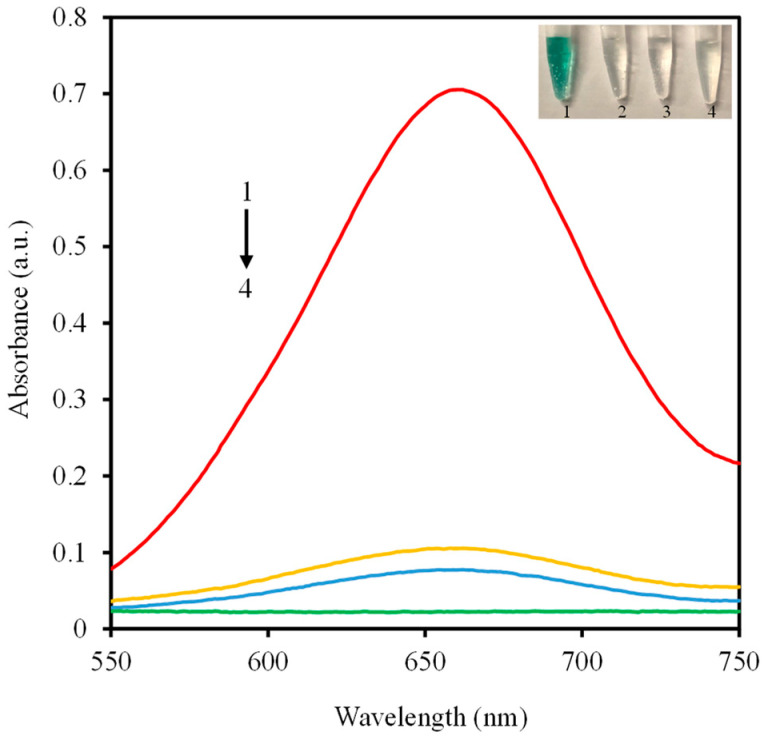
UV-Vis absorption spectra and digital photograph of VB6-TMB-H_2_O_2_ (1), VB6-TMB (2), TMB-H_2_O_2_ (3) and VB6-H_2_O_2_ (4).

Figure 1 depicts the enzymatic activity of VB6 by catalyzing TMB at the existence of H_2_O_2_. Generally, the typical peroxidase substrate colorless TMB can be catalytically oxidized to blue product TMB oxide, which exhibits obvious absorption peak at 652 nm correspondingly. As shown in Figure 2, there is neither apparent absorption at 652 nm nor color variance of resultant solutions when VB6, TMB, and H_2_O_2_ were incubated in pairs, respectively. In contrast, the absorbance intensity and color of reaction system containing TMB and H_2_O_2_ is obviously boosted with the addition of VB6, which verifies the peroxidase-like catalytic activity of VB6.

### 2.2. Optimization of Reaction Conditions

**Figure 3:**
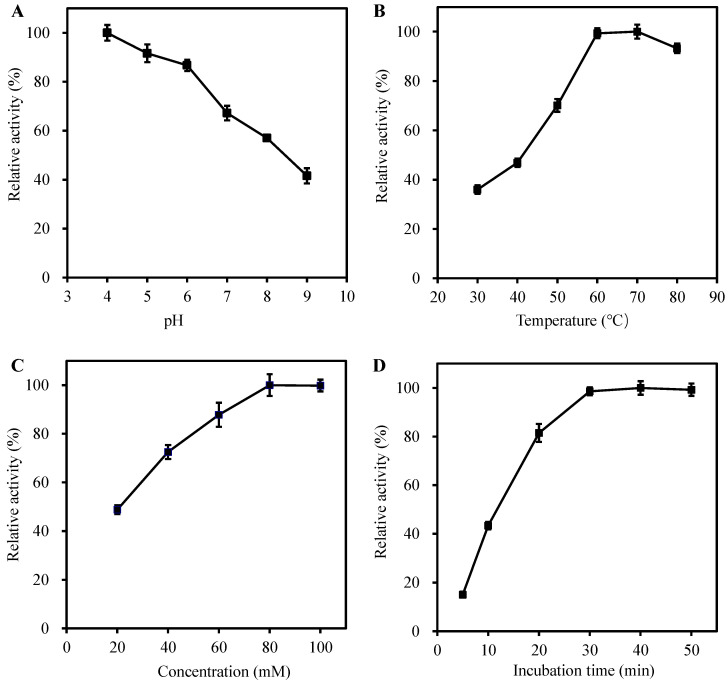
Effects of (**A**) pH, (**B**) temperature, (**C**) VB6 concentration, and (**D**) incubation time on the peroxidase-like activity of VB6. Experiments were carried out using 100 mΜ of VB6 dissolved in PBS buffer (10 mM, pH = 4.0) at 60 °C with 4.0 mΜ of TMB and 0.5 mM of H_2_O_2_ as substrates. The maximum point in each curve is set as 100%. The error bars represent the standard deviations derived from three independent measurements.

The peroxidase-like activity of VB6 is easily influenced by the pH, temperature, VB6 concentration, and incubation time. Therefore, the factors that will influence the peroxidase-like property of VB6 were investigated. As depicted in Figure 3A, like most of other peroxidase mimics, the catalytic activity of VB6 decreases under neutral or alkaline conditions and possesses best peroxidase-like activity at pH 4.0. In addition, the temperature-dependent peroxidase-like activity (Figure 3B) demonstrates that the optimal temperature is 60 °C, and the catalytic activity of VB6 keeps relative stable under high temperature, which may be ascribed to the good thermostability of VB6. Furthermore, results shown in Figure 3C,D indicate that when the VB6 concentration and incubation time were selected as 80 mM and 30 min, respectively, the peroxidase-like activity of VB6 reaches a plateau. Therefore, pH 4.0, 60 °C, 80 mM of VB6 and 30 min of incubation time were selected as the optimal reaction conditions to carry out subsequent analysis.

### 2.3. Kinetic Analysis

To further delve into the peroxidase-like catalytic activity of VB6, the effect of varied TMB concentrations with fixed H_2_O_2_ concentration or vice versa on the steady-state kinetics was investigated under optimal conditions. The results shown in Figure S1a–d confirm typical Michaelis–Menten curves of TMB and H_2_O_2_, from which the Michaelis–Menten constant (*K_m_*) and maximal reaction velocity (*V_max_*) were calculated. Notably, *K_m_* is regarded as a significant indicator of the affinity between the enzyme and substrate, and a lower value represents higher affinity. As summarized in Table S1, the *K_m_* of VB6 and H_2_O_2_ is 0.25 mM, which is approximately five times smaller than natural horseradish peroxidase and some other small-molecule and nanomaterials-based peroxidase simulants, indicating the superiority of VB6 in attracting H_2_O_2_ over other previously reported peroxidase mimics. In addition, the *K_m_* value of VB6 taking TMB as substrate is 5.33 mM, indicating only a small amount of TMB is needed to reach the maximum catalytic activity. However, both *V_max_* values of VB6 taking H_2_O_2_ or TMB as substrate are lower than that of HRP. Therefore, it is still a challenge to excavate small-molecule peroxidase mimics with higher catalytic performance.

### 2.4. Catalytic Mechanism Investigation

Generally, hydroxyl radicals (•OH) and superoxide radicals (O_2_•^−^) are the possible dominant active species responsible for the catalytic activity. To investigate the possible catalytic mechanism of the peroxidase-like activity of VB6, the radical trapping assay by introducing different radical scavengers has been performed to investigate the main active species in the catalytic reaction system. As shown in Figure S2, with the increase in introduced amount of •OH scavenger isopropanol, there exhibits an obvious decline in the relative activity of the VB6-catalyzed reaction system, which indicates that •OH is accounted for the catalytic process. However, when increased amount of O_2_•^−^ scavenger superoxide dismutase (SOD) was added into the reaction system, no apparent absorbance variance was observed, which demonstrates the negligible role of O_2_•^−^ in the catalytic reaction. Therefore, the catalytic mechanism of VB6 as a peroxidase mimic can be ascribed to the •OH produced from H_2_O_2_ decomposition, and then •OH reacts with TMB to obtain blue oxTMB product.

### 2.5. Determination of H_2_O_2_

**Figure 4:**
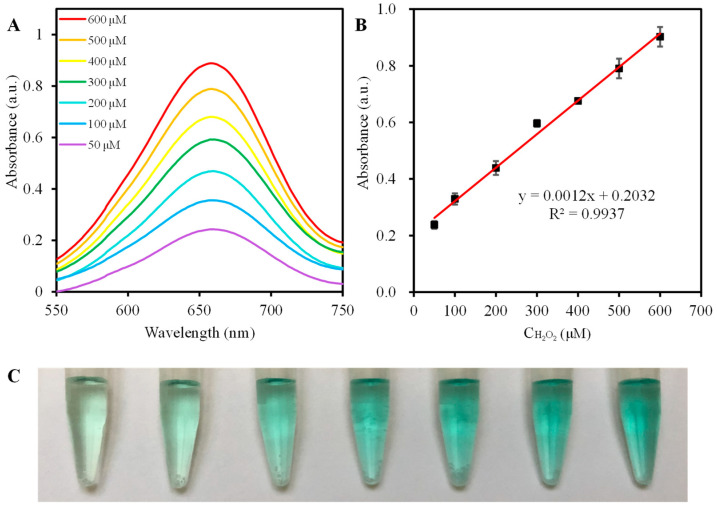
(**A**) The UV-Vis absorption spectra of VB6-TMB system upon adding different concentrations of H_2_O_2_ (50.0–600.0 μM, from bottom to top); (**B**) Calibration plot of the absorbance versus the concentrations of H_2_O_2_ under the optimal conditions; (**C**) The corresponding digital photograph. The error bars represent the standard deviations of three independent measurements.

Considering the intrinsic peroxidase-like activity of VB6, a facile VB6-TMB colorimetric detection method for H_2_O_2_ determination was fabricated. Figure 4A,B demonstrate the H_2_O_2_ concentration-response plot for the absorbance of the reaction mixture with varied H_2_O_2_ concentrations in the range of 50–600 μM, which intuitively represents that the absorbance at 652 nm is enhanced as the H_2_O_2_ concentration increased. The corresponding linear regression equation is A = 0.0012 [H_2_O_2_] + 0.2032 (R^2^ = 0.9937). The limit of detection (LOD) is determined to be 12.1 μM. In Table 1, as compared with other methods based on the nanomaterials-based peroxidase mimics, the convenient colorimetric method utilizing VB6-TMB reaction system has the merit of a wider linear range and a lower LOD value. In addition, as shown in Figure 4C, the color change of the VB6-TMB reaction system can be easily distinguished by naked eyes. Therefore, the results illustrate that VB6-TMB is promising and feasible to be used for H_2_O_2_ determination in real samples.

Generally, H_2_O_2_ is extensively applied as a preservative agent in commercial milk, which may take a toll on human health when excessive H_2_O_2_ residue exists in milk. Therefore, the detection of residual H_2_O_2_ amount in commercial milk samples is meaningful. To estimate the applicability of the VB6-TMB based H_2_O_2_ detection method, the recovery of H_2_O_2_ by the standard addition in three brands of milk samples were measured. As shown in Table S2, the recovery of H_2_O_2_ in commercial milk samples are in the range of 95.2% to 105.4%, and the relative standard deviations (RSD) are between 2.7% and 7.2%. Thus, the developed colorimetric method shows high accuracy in H_2_O_2_ detection.

### 2.6. Gallic Acid Determination

**Figure 5:**
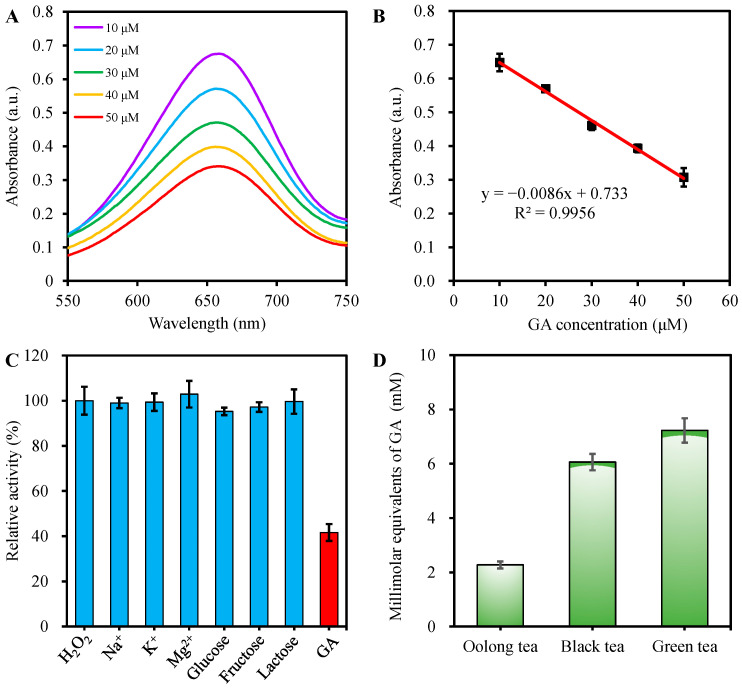
(**A**) The absorbance spectra of VB6-TMB-H_2_O_2_ catalytic system at the existence of different concentrations of gallic acid and (**B**) the linear calibration plot of absorbance against concentrations of gallic acid; (**C**) Selectivity of gallic acid determination with interferences by recording the signal response at 652 nm, including 0.05 mM of gallic acid and 2.5 mM of interferences (K^+^, Na^+^, Mg^2+^, lactose, fructose, and glucose); (**D**) The total antioxidant capacity evaluation of different tea infusions with gallic acid equivalent as indicator. The error bars represent the standard deviations of three independent measurements.

The strong reduction ability of GA can convert the oxTMB to colorless TMB, a simple colorimetric GA detection method based on the absorbance decline in the VB6-TMB system was fabricated. As depicted in Figure 5A,B, with the increase in GA concentration, the absorbance intensity of the mixture at 652 nm declines gradually. Furthermore, there is a good linear correlation between absorbance values at 652 nm and GA concentrations within the range of 10.0–50.0 μM. The linear regression equation is A = −0.0086 [GA] + 0.733 (R^2^ = 0.9956), and the LOD for GA is determined to be 4.1 μM. Compared with other methods used for GA detection based on nanomaterials-related peroxidase simulants (Table 2), this colorimetric method has the advantages of a wide linear range and no requirement of harsh synthesis conditions.

The selectivity and interference assay for GA determination was performed at the coexistence of some common interference such as metal ions (Na^+^, K^+^, and Mg^2+^) and sugars (lactose, fructose, and glucose) under optimal conditions. Figure 5C indicates that only the introduction of GA gives rise to a significant reduction of the signal response and negligible absorbance intensity variance is observed even if the interference concentration is 50 times higher than that of GA. These results prove the good selectivity and promising applicability for the GA determination in practical samples.

Generally, total antioxidant capacity (TAC) is a significant indicator for evaluating the quality of some foods and drugs with antioxidant functions. To further evaluate the applicability of the developed method, it was implemented to evaluate the TAC capacity in different tea infusions. GA is an important natural phenolic compound, which exhibits strong antioxidant activity and naturally exists in various tea [28]. In this work, TAC capacity was measured as millimolar equivalents of GA [29]. Before analysis, the tea infusions were diluted by appropriate multiples to conform to the linear range of standard calibration plot. As shown in Figure 5D, based on the calibration plot, the millimolar equivalents of GA concentrations of Oolong tea, black tea, and green tea obtained from commercial are calculated to be 2.3, 6.1, and 7.2 mM, respectively. In addition, the RSD values are within 5.8 to 6.3%, which confirms the good precision of the TAC evaluation by the developed method.

**Table 1:** Comparisons of colorimetric detection of H_2_O_2_ by various peroxidase mimics.

Enzyme Mimics	Methods	Linear Range (μM)	LOD (μM)	Ref.
Cu_2_(OH)_3_Cl-CeO_2_	Colorimetric	20–50	10	[30]
MMT-CeO_2_	Colorimetric	9–500	7.8	[31]
CoS	Colorimetric	50–800	20	[32]
GO-FeTPyP	Colorimetric	20–500	72	[33]
Fe-Ag_2_S	Colorimetric	10–150	7.82	[34]
Fe_3_O_4_@AuNPs	Colorimetric	40–5500	11.1	[35]
Zr-MOF-PVP	Colorimetric	10–800	2.16	[36]
VB6	Colorimetric	50–600	12.1	This work

Cu_2_(OH)_3_Cl-CeO_2_: A nanomaterial of the chemical composition Cu_2_(OH)_3_Cl-CeO_2_; MMT-CeO_2_: CeO2-montmorillonite (MMT) nanocomposites; GO-FeTPyP: supramolecular assemblies of tetrapyridylporphyrin (TPyP) and its metallic complexes with graphene oxide (GO); Fe-Ag_2_S: Fe-doped Ag_2_S nanocomposites; Fe_3_O_4_@AuNPs: AuNPs distributed on the surface of Fe_3_O_4_ particles; Zr-MOF-PVP: Zr-based MOFs capped with polyvinylpyrrolidone; VB6: vitamin B6.

**Table 2:** Comparisons of determination of gallic acid based on different peroxidase mimics.

Sensors	Methods	Linear Range (μM)	LOD (μM)	Ref.
Poly-Glu/rGO	Electrochemistry	1–17	0.53	[37]
CPE/MWNT	Electrochemistry	0.5–15	0.3	[38]
CDs/CoOOH	Fluorescence	2–60	1.2	[39]
NaErF_4_:Tm@SiO_2_@ZIF-8	Fluorescence	0–30	0.35	[40]
LaFeO_3_	Colorimetric	0.6–36	0.4	[41]
VB6	Colorimetric	10–50	4.1	This work

Poly-Glu/rGO: the electro-polymerisation of glutamic acid on reduced graphene oxide (rGO) modified paraffin impregnated graphite electrode; CPE/MWNT: CPE-carbon paste electrode, MWNT-multiwalled carbon nanotube; CDs/CoOOH: Carbon dots/CoOOH nanoflakes; NaErF_4_: Tm@SiO_2_@ZIF-8: a core-shell structured upconversion nanoparticles@zeolitic imidazolate frameworks (ZIF-8) fluorescent nanoprobe; LaFeO_3_: Perovskite mesoporous lanthanum ferrite (LaFeO_3_); VB6: vitamin B6.

## 3. Materials and Methods

### 3.1. Chemicals and Materials

Pyridoxine hydrochloride (VB6), D(+)-Lactose, D(+)-Glucose, and superoxide dismutase (SOD) were obtained from Yuanye Biological Technology Co., Ltd. (Shanghai, China). TMB was purchased from Titan Scientific Co., Ltd. (Shanghai, China). Anhydrous ethanol was bought from Chongqing Chuandong Chemical Co., Ltd. (Chongqing, China). H_2_O_2_ (30% in water), isopropanol, fructose, sodium chloride, magnesium sulfate heptahydrate, and potassium chloride were obtained from Chengdu Chron Chemicals Co., Ltd. (Chengdu, China). The Oolong tea was purchased from Chongqing Yinrong Trading Co., Ltd. (Chongqing, China). The black tea and green tea were purchased from Unilever Food Co., Ltd. (Shanghai, China). The water used in all experiments was purified by a water purification system (ATSelem 1820A, Antesheng Environmental Protection Equipment Co., Ltd., Chongqing, China).

### 3.2. Peroxidase-like Catalytic Activity of VB6

The peroxidase-like catalytic activity of VB6 was validated through a typical oxidation reaction of chromogenic substrate TMB in the presence of H_2_O_2_. In detail, VB6 (80 mM) was mixed homogenously with TMB (4 mM) and H_2_O_2_ (0.5 mM), and the reaction system was incubated at 60 °C for 30 min. Subsequently, the absorbance (intensity) at 652 nm of the blue solution due to the oxidation of TMB was determined by a UV-5500 UV/VIS spectrophotometer (Metash, Shanghai, China).

### 3.3. Kinetic Tests of VB6

The kinetic tests of the peroxidase-like catalytic reaction were carried out by fixing the concentration of one substrate (H_2_O_2_ or TMB) while changing the concentration of another. The reaction systems were incubated at 60 °C for 30 min and the absorbance of reaction mixture were recorded. Then, the kinetic parameters, including *v_max_* and *K_m_*, were determined through plotting the Lineweaver–Burk curve.

*ν* = *v_max_* [*S*]/(*K_m_* + [*S*]) (1)

where *ν* is the initial reaction rate, *v_max_* represents the maximum reaction rate, *K_m_* indicates the Michaelis–Menten constant, and [*S*] denotes substrate concentration.

### 3.4. Detection of H_2_O_2_ and GA

Taking advantage of the excellent peroxidase-like activity of VB6, a facile colorimetric assay for H_2_O_2_ and GA determination was fabricated. The detection of H_2_O_2_ was performed according to the following steps. The absorbance intensity of the reaction system containing 80.0 mM of VB6, 4.0 mM of TMB, and varied concentrations of H_2_O_2_ (0–0.5 mM) were recorded at 652 nm after incubation at 60 °C for 30 min. To verify the feasibility of H_2_O_2_ detection in real samples, three different brands of milk were selected as practical specimens. Prior to the assay procedure, 3.0 mL of each sample was centrifuged at 6745× *g* for 15 min by three times to achieve protein and fat elimination. Subsequently, the clear filtrate of each sample was diluted 10 multiples with phosphate buffer.

The detection of GA was carried out according to the following steps. In brief, 80.0 mM of VB6, 4.0 mM of TMB, 0.5 mM of H_2_O_2_ and different concentrations of GA solutions were homogeneously blended and incubated at 60 °C for 30 min, and the absorbance intensity at 652 nm of the resultant solution was measured for GA detection. To validate the applicability of GA determination in real samples, three kinds of tea were chosen as actual samples. Before analysis, each sample of Oolong tea, black tea, and green tea (1.0 g) was soaked with boiling pure water (20.0 mL) three times, and then the tea infusion was collected together, followed by filtration through 0.22 μm filter membranes. Subsequently, the infusion of each tea was diluted with phosphate buffer by appropriate multiples to fit the linear range of the calibration plot.

The limit of detection (LOD) was calculated by the following equation: LOD = 3*δ*/*k*, where *δ* is the standard deviation of blank measurement, and *k* is the slope between the absorbance of solution versus H_2_O_2_ or GA concentration.

## 4. Conclusions

In summary, this work firstly demonstrates the inherent peroxidase-like activity of VB6, which is favorable of H_2_O_2_ decomposition to generate •OH and further catalyze the oxidation of colorless TMB to the blue product oxTMB. Furthermore, the kinetic assay indicates that the affinity between H_2_O_2_ and VB6 is higher than that of natural horseradish peroxidase. Based on this discovery, a simple colorimetric method for the determination of H_2_O_2_ and GA was successfully established with a wide linear range and low detection limit. Compared with most of other nanomaterials-based peroxidase simulants and natural horseradish peroxidase, VB6 has the advantages of superior biocompatibility, higher stability, and good catalytic activity at a high temperature and no requirement of complicated preparation processes. Therefore, VB6 has a broad application prospect in analysis fields. Besides, this study may contribute to the profound insight of the physiological functions of VB6 and lay foundation for further excavation of more small-molecule peroxidase mimics.

## Data Availability

The data presented in this study are contained within the article.

## Supplementary Materials

The following supporting information can be downloaded at: https://www.mdpi.com/article/10.3390/molecules27134262/s1, Figure S1: Reaction velocity under 4.0 mM of TMB with varied concentrations of H_2_O_2_ (A) and the corresponding double-reciprocal plots of VB6-catalyzed activity (B); Reaction velocity under 0.5 mM of H_2_O_2_ with varied concentrations of TMB (C) and the corresponding double-reciprocal plots of VB6-catalyzed activity (D). Error bars represent the standard deviation of three independent measurements; Figure S2: Effects of various active scavengers during the catalysis of TMB by VB6; Table S1: Kinetic parameters (*K_m_* and *v_max_*) of different small-molecule and nanomaterials-based peroxidase mimics; Table S2: H_2_O_2_ detection in different brands of milk samples [21,22,23,32,42].

## Sample Availability

Samples of the compounds are available from the authors.
