# Supplementary material for: Investigation on the Peroxidase-like Activity of Vitamin B6 and Its Applications in Colorimetric Detection of Hydrogen Peroxide and Total Antioxidant Capacity Evaluation
Source: Molecules. 2022 Jul 1;27(13):4262. doi: 10.3390/molecules27134262 (PMC9268325; doi:10.3390/molecules27134262)
Supplement: Supplementary file 1 [file molecules-27-04262-s001.zip › molecules-1757360-supplementary.pdf]

Electronic Supplementary Material

Investigation on the Peroxidase-Like Activity of Vitamin B6 and Its Applications in Colorimetric Detection of Hydrogen Peroxide and Total Antioxidant Capacity Evaluation

Chun-Yan Zhang, Li-Jing Peng, Guo-Ying Chen, Hao Zhang, Feng-Qing Yang*

School of Chemistry and Chemical Engineering, Chongqing University, Chongqing 401331, China

*Corresponding author:

Prof. Dr. Feng-Qing Yang, School of Chemistry and Chemical Engineering, Chongqing University, Chongqing 401331, China.

Tel: +8613617650637. E-mail: fengqingyang@cqu.edu.cn.

Table of contents

Supplementary Figures

Figure S1. Reaction velocity under 4.0 mM of TMB with varied concentrations of H_2O_2 (A) and the corresponding double-reciprocal plots of VB6-catalyzed activity (B); Reaction velocity under 0.5 mM of H_2O_2 with varied concentrations of TMB (C) and the corresponding double-reciprocal plots of VB6-catalyzed activity (D). Error bars represent the standard deviation of three independent measurements.

Figure S2. Effects of various active scavengers during the catalysis of TMB by VB6.

Supplementary Tables

Table S1. Kinetic parameters (K_m and v_{max}) of different small-molecule and nanomaterials-based peroxidase mimics.

Table S2. H_2O_2 detection in different brands of milk samples.

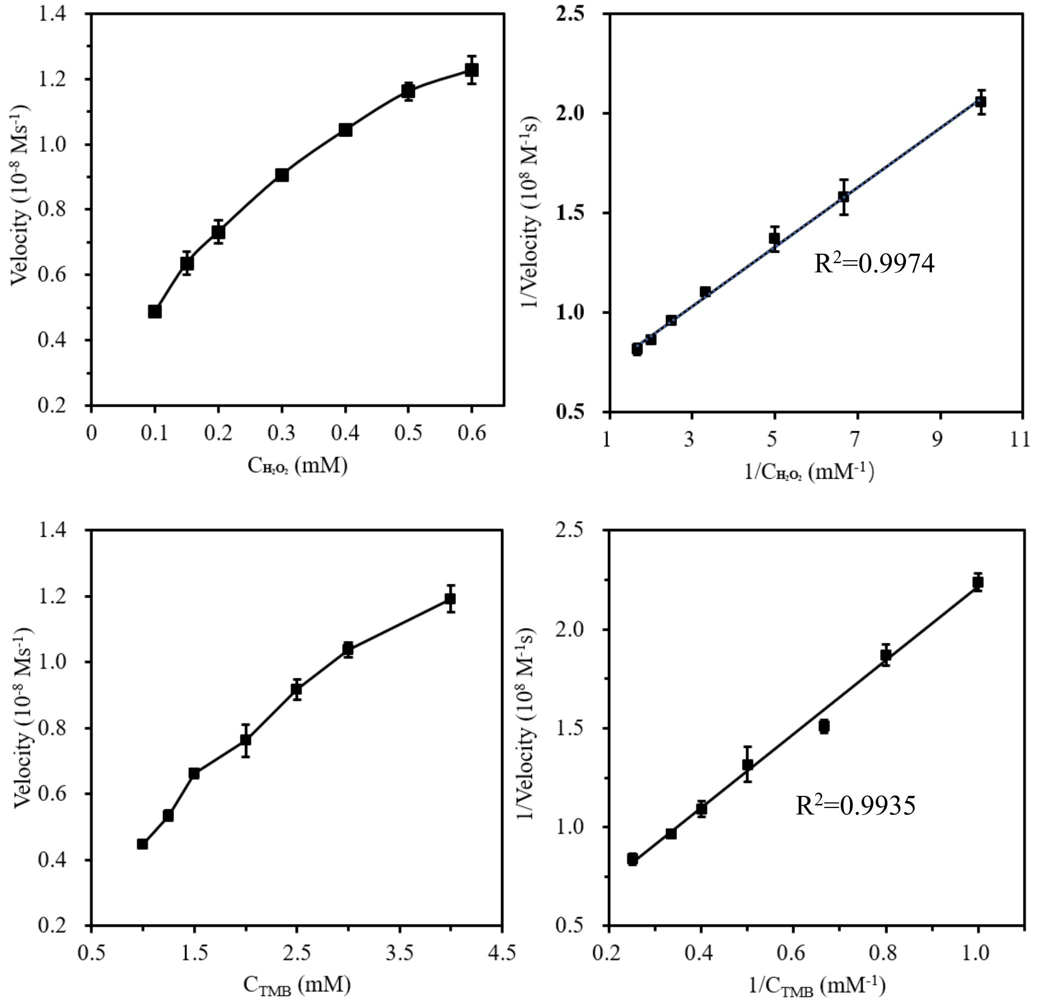

Figure S1. Reaction velocity under 4.0 mM of TMB with varied concentrations of H_2O_2 (A) and the corresponding double-reciprocal plots of VB6-catalyzed activity (B); Reaction velocity under 0.5 mM of H_2O_2 with varied concentrations of TMB (C) and the corresponding double-reciprocal plots of VB6-catalyzed activity (D). Error bars represent the standard deviation of three independent measurements.

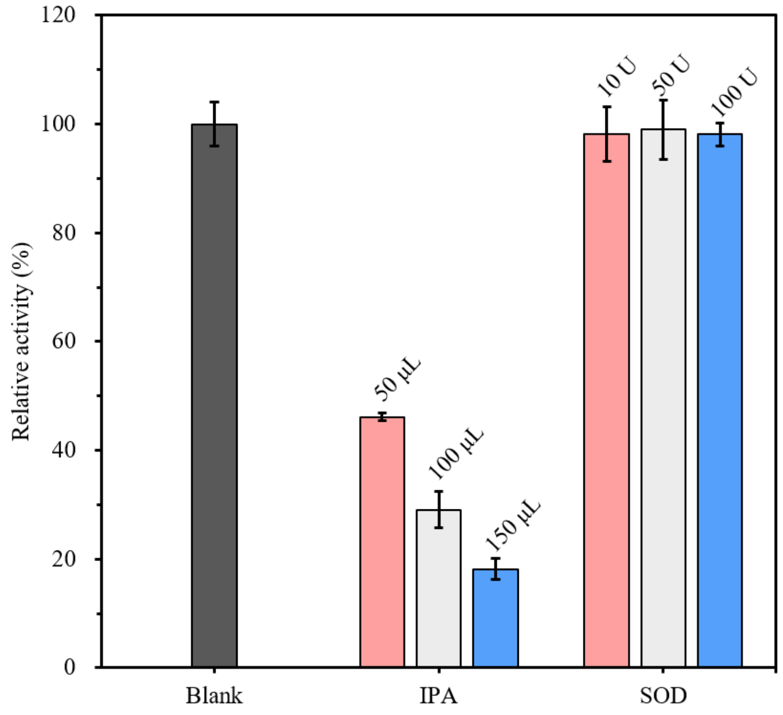

Figure S2. Effects of various active scavengers during the catalysis of TMB by VB6.

Table S1. Kinetic parameters (K_m and v_{max}) of different small-molecule and nanomaterials-based peroxidase mimics.

Catalyst	K_m (mM)		v_{max} (10^{-8} M s^{-1})		Ref.
	TMB	H_2O_2	TMB	H_2O_2	
AF	1.90 ± 0.126	1.86 ± 0.232	0.384 ± 0.16	0.217 ± 0.02	[21]
DFF	1.78	2.97	0.404	0.983	[22]
PA/$Cu_3(PO_4)_2$·$3H_2O$	3.6	4.0	1.6	1.3	[42]
CoS	0.41	7.15	5.82	2.65	[32]
HRP	0.179 ± 0.020	1.18 ± 0.147	3.895 ± 0.218	7.307 ± 0.824	[23]
VB6	5.33	0.25	2.85	1.71	This work

K_m: Michaelis constant; v_{max}: the maximal reaction rate; AF: Aminofluorescein; DFF: 2′, 7′-difluorofluorescein; PA/$Cu_3(PO_4)_2$·$3H_2O$: hydrophilic phytic-acid modified copper phosphate; CoS: sphere-like cobalt sulfide with nanostructures; HRP: Horseradish peroxidase; VB6: Vitamin B6, pyridoxine hydrochloride.

Table S2. H_2O_2 detection in different brands of milk samples.

Samples	Added (μM)	Found (μM)	Recovery (%)	RSD (%)
	0	0	-	-
Tianyou	100.0	103.4	103.4	6.9
	300.0	297.0	99.0	5.3
	0	0	-	-
Yili	100.0	102.9	102.9	6.5
	300.0	294.0	98.0	7.2
	0	0	-	-
Mengniu	100.0	105.4	105.4	5.8
	300.0	285.7	95.2	2.7
